# *Candida auris* and COVID-19: A health threatening combination

**DOI:** 10.18502/cmm.8.3.11211

**Published:** 2022-09

**Authors:** Shaghayegh Khojasteh, Jalal Jafarzdeh, Seyed Abdollah Hosseini, Iman Haghani, Habibollah Turki, Sanaz Aghaei Gharehbolagh, Mahdi Abastabar, Shahram Mahmoudi

**Affiliations:** 1 Department of Medical Mycology, School of Medicine, Mazandaran University of Medical Sciences, Sari, Iran; 2 Invasive Fungi Research Center, Communicable Diseases Institute, Mazandaran University of Medical Sciences, Sari, Iran; 3 Molecular Medecine Research Center, Hormozgan Health Institute, Hormozgan University of Medical Sciences, Bandar Abbas, Iran; 4 Department of Medical Mycology and Parasitology, School of Medicine, Babol University of Medical Sciences, Babol, Iran; 5 Toxoplasmosis Research Center, Communicable Diseases, Institute, Mazandaran University of Medical Sciences, Mazandaran, Sari, Iran; 6 Department of Medical Parasitology and Mycology, School of Public Health, Tehran University of Medical Sciences, Tehran, Iran; 7 Department of Parasitology and Mycology, School of Medicine, Iran University of Medical Sciences, Tehran, Iran

**Keywords:** Antifungal resistance, *Candida auris*, Co-infection, COVID-19, SARS-CoV-2

## Abstract

Since its first emergence in December 2019, due to its fast distribution throughout the world, SARS-COV-2 become a global concern. With the extremely increased number of hospitalized patients, this situation provided a potential basis for the transmission of nosocomial infections. *Candida auris* is a multidrug-resistant pathogen with improved transmission dynamics and resistance traits. During the worldwide spread of COVID-19, cases or outbreaks of *C. auris* colonization or infection have been reported. Resistance to antifungal drugs has been observed in the causative agents of the majority of such cases. The focus in this review is on COVID-19-associated *C. auris* infections (case studies/outbreaks) and the pandemic's potential effect on antifungal drug resistance.

## Introduction

Severe acute respiratory syndrome coronavirus 2 (SARS-CoV-2), the most significant global health event since Spanish influenza in the early 20th century, is alarmingly on the rising and threatens human health and public safety [ 1 , 2 ]. Unlike influenza outbreaks, coronavirus disease 2019 (COVID-19) has spread fast all over the world, and over 100 countries have reported cases of this disease [ 1 , 3 , 4 ]. SARS-CoV-2 ranks third among members of the Coronavirus family regarding its pathogenicity; however, due to its rapid spreading, it has posed the severest threat to global health in this century [ 1 ]. 

The hospital mortality of COVID-19 is estimated to range from 15% to 20% and increases to 40% among patients requiring intensive care unit (ICU) admission [ 5 ]. Meanwhile, early estimates suggested that the true burden of disease and an actual number of deaths may be as much as 10 times higher than reported cases [ 4 , 6 , 7 ]. Patients with severe COVID-19 need intensive care, including mechanical ventilation, extracorporeal membrane oxygenation, continuous renal replacement therapy, glucocorticoids, and intravenous immune-globulin therapy. These interventions could predispose patients to co-infections by different microorganisms including fungi (both filamentous fungi and yeasts) [ 8 - 10 ]. Co-infections by *Candida auris*, due to its persistence on hospital surfaces and high resistance to antifungal drugs, are of significant value, and COVID-19 has provided a potential bed for these infections [ 11 , 12 ]. Patients admitted to ICU have the greatest risk factors for such infections [ 11 , 13 ]. 

Antimicrobial resistance (AMR) as another threat to global health and the economy is likely to be overshadowed by the COVID-19 pandemic [ 2 ]. Currently, infections caused by antimicrobial-resistant pathogens are responsible for nearly 700,000 deaths every year worldwide. It can be anticipated that AMR-related deaths due to the catastrophe status of the COVID-19 pandemic can reach up to 10 million deaths per year by 2050 if the world could not tackle these current states [ 14 , 15 ]. 

So far, cases or outbreaks of *C. auris* infection/colonization among COVID-19 patients have been reported [ 16 - 18 ]. In this study, we have a particular focus on the *C. auris* infection/colonization in patients with COVID-19 and the potential impact of this viral pandemic on antifungal drug resistance.

## Candida auris in the era of COVID-19

*C. auris*, first isolated in Japan in 2009, is an emerging member of the Metschnikowiaceae family within the *Candida/Clavispora* clade [ 19 ]. To date, *C. auris* has been reported from at least 40 countries; therefore, it has a global distribution [ 11 , 20 , 21 ]. *C. auris* has been isolated as an infecting or colonizing agent from various specimens or parts of the human body including blood, urine, wounds, bile, the nares, the skin, the axilla, and the rectum of patients [ 22 , 23 ]. Furthermore, this fungus can survive on environmental surfaces and human skin for several weeks and can even tolerate some frequently used disinfectants [ 24 - 26 ]. These traits can be associated with intrahospital transmission of *C. auris*, leading to outbreaks [ 27 , 28 ]. In the past decade, *C. auris* has led to several outbreaks in hospitals worldwide and become a global health threat [ 29 ]. Invasive infections by this pathogen are usually observed in critically ill patients in ICUs and are related to high mortality rates [ 30 ].

COVID-19 has presented a great challenge for health care settings. During this viral pandemic, patients admitted to ICUs are at the greatest risk for *C. auris* infection/colonization [ 31 ]. In the second half of 2020, several countries, such as India, Lebanon, Italy, Brazil, Guatemala, Mexico, Peru, Panama, Colombia, and the United States, reported cases/outbreaks of co-infection by *C. auris* in COVID-19 patients. [ 32 - 35 ]. Accordingly, attention should be drawn to this topic to characterize various features of these co-infections. By a literature review up to September 12, 2022, 27 studies were found, of which data of COVID-19-associated *C. auris* infections were extractable in 14 studies (75 cases, Table 1). 

**Table 1:** Characteristics of patients with COVID-19-associated *Candida auris* infections reported up to September 12, 2022

Ref.	Publication Year	Country	Sex	Age	Underlying Conditions	Risk Factors	Clade (I,II,III,IV,V)	Site of infection OR Colonization	Resistance Pattern	Hospital stay (day)	Antifungal Treatment	Outcome
[ 12 ]	2020	Mexico	M	51	HT, DS, Obesity	MV, PICCs, UC, Antibiotic use, Steroid therapy	IV	Blood	AMB, FLC	20-70	CAS, ANF	Died
[ 12 ]	2020	Mexico	M	54	HT, DS, Obesity, Asthma	MV, PICCs, UC, Antibiotic use, Steroid therapy,	IV	Urine	AMB	20-70	ISA, CAS	Survived
[ 12 ]	2020	Mexico	M	55	HT, DS, CAD	MV, PICCs, UC, Antibiotic use, Steroid therapy	IV	Blood	AMB	20-70	ANF	Died
[ 12 ]	2020	Mexico	M	51	Obesity	MV, PICCs, UC, Antibiotic use, Steroid therapy	IV	Urine	AMB	20-70	ISA, ANF	Died
[ 12 ]	2020	Mexico	M	64	AKD	MV, PICCs, UC, Antibiotic use, Steroid therapy	IV	Blood, Urine	AMB, FLC	20-70	CAS, VRC, AMB	Died
[ 12 ]	2020	Mexico	M	64	HT, Smoking, Obesity, Hypothyroidism	MV, PICCs, UC, Antibiotic use, Steroid therapy	IV	Blood, PIC line, Urine	AMB	20-70	ANF, ISA	Died
[ 12 ]	2020	Mexico	F	54	HT, Obesity	MV, PICCs, UC, Antibiotic use, steroid therapy	IV	Blood	AMB, FLC	20-70	AMB, CAS, VRC	Died
[ 12 ]	2020	Mexico	F	60	Obesity	MV, PICCs, UC, Antibiotic use, Steroid therapy,	IV	Urine	AMB	20-70	CAS, ANF, VRC	Died
[ 12 ]	2020	Mexico	M	58	HT, Obesity	MV, PICCs, UC, Antibiotic use, Steroid therapy	IV	Urine	AMB, FLC	20-70	ANF	Died
[ 12 ]	2020	Mexico	M	36	DS, Obesity	MV, PICCs, UC, Antibiotic use, Steroid therapy	IV	Urine	AMB, FLC	20-70	CAS	Survived
[ 12 ]	2020	Mexico	M	66	HT, DS, CAD, VHD	MV, PICCs, UC, Antibiotic use, Steroid therapy,	IV	Urine	AMB, ANF	20-70	VRC, CAS	Survived
[ 12 ]	2020	Mexico	M	46	Obesity	MV, PICCs, UC, Antibiotic use, Steroid therapy	IV	Blood	AMB	20-70	VRC, CAS	Survived
[ 42 ]	2020	USA	F	49	Seizure disorder	ND	ND	Blood	ND	14	MFG	Survived
[ 33 ]	2020	India	F	25	CLD, AKD	Antibiotic use, CVC, UC	ND	Blood	FLC, VOR, 5-FC	35	AMB	Survived
[ 33 ]	2020	India	M	52	HT, DS	Antibiotic use, Steroid therapy, CVC, and UC	ND	Blood	FLC	20	MFG, AMB	Died
[ 33 ]	2020	India	F	82	HT, DS, Hypothyroidism, CKD	Antibiotic use, Steroid therapy, CVC, UC	ND	Blood	FLC	60	MFG	Died
[ 33 ]	2020	India	F	86	CLD, IHD, DS	Antibiotic use, Steroid therapy, CVC, UC	ND	Blood	FLC	21	MFG	Died
[ 33 ]	2020	India	M	66	HT, DS, asthma	Antibiotic use, CVC, UC	ND	Blood	FLC, AMB	20	MFG, AMB	Survived
[ 33 ]	2020	India	M	71	Hypothyroidism, CKD	Antibiotic use, Steroid therapy, CVC, UC	ND	Blood	FLC, 5-FC	32	MFG	Died
[ 33 ]	2020	India	M	67	HT, DS, COPD	Antibiotic use, steroid therapy, CVC, and UC	ND	Blood	FLC, AMB, 5- FC	21	MFG, AMB	Survived
[ 33 ]	2020	India	M	72	HT, CLD	Antibiotic use, Steroid therapy, CVC, UC	ND	Blood	FLC, VOR, AMB, 5-FC	27	MFG	Died
[ 33 ]	2020	India	M	81	HT, DS, IHD	Antibiotic use, Steroid therapy, CVC, UC	ND	Blood	FLC, VOR, 5- FC	20	MFG	Died
[ 33 ]	2020	India	M	69	HT, Asthma	Antibiotic use, Steroid therapy, CVC, UC	ND	Blood	FLC, AMB, 5- FC	21	MFG	Survived
[ 34 ]	2021	Italy	M	70	DS, Obesity	ND	ND	BAL (BSI)	AMB, azoles	ND	ND	Died
[ 34 ]	2021	Italy	M	62	None	Antibiotic use	ND	Surveillance swab (BSI)	AMB, azoles	48	CAS	Survived
[ 34 ]	2021	Italy	M	69	CAD	Antibiotic use	ND	Surveillance swab (BSI)	AMB, azoles	26	AMB, CAS	Died
[ 34 ]	2021	Italy	M	50	None	Antibiotic use	ND	Surveillance swab	AMB, azoles	ND	ND	Survived
[ 34 ]	2021	Italy	M	62	HT	Antibiotic use	ND	BAL (BSI)	AMB, azoles	24	CAS	Survived
[ 34 ]	2021	Italy	M	64	Asthma, HT	Antibiotic use	ND	Blood (BSI)	AMB, azoles	29	CAS	Died
[ 41 ]	2021	Italy	ND	ND	ND	ND	I	BAL	AMB, VRC, FLC	ND	ND	Died
[ 41 ]	2021	Italy	ND	ND	ND	ND	I	BAL	AMB, VRC, FLC	ND	ND	Survived
[ 41 ]	2021	Italy	ND	ND	ND	ND	I	Blood	AMB, VRC, FLC	ND	ND	Died
[ 41 ]	2021	Italy	ND	ND	ND	ND	I	BAL	AMB, VRC, FLC	ND	ND	Died
[ 41 ]	2021	Italy	ND	ND	ND	ND	I	Urine	AMB, VRC, FLC	ND	ND	Survived
[ 36 ]	2021	Brazil	M	59	DVT	MV, HD, Steroid therapy	I	CVC-tip	MDS	49	ANF	Survived
[ 36 ]	2021	Brazil	F	74	CKD, DS, HT	DVT , Noninvasive ventilation, HD, Steroid therapy, Antibiotic use, HD	I	Blood	MDS	70	ANF	Died
[ 40 ]	2021	USA	M	72	DLP	MV, Use of vasopressor agents, Antecedent Steroid therapy, Antibiotic use	III	Blood	Echino, FLC	14	MFG	Survived
[ 40 ]	2021	USA	M	77	DS, HT, DLP	MV, Use of vasopressor agents, Antecedent Steroid therapy, Antibiotic use	III	Urine	FLC	28	ND	Died
[ 40 ]	2021	USA	F	71	MM, SCT	MV, Use of vasopressor agents, Antecedent Steroid therapy, Antibiotic use	III	Blood	FLC	24	MFG, AMB	Died
[ 40 ]	2021	USA	M	71	DS, HT	MV, Use of vasopressor agents, Antecedent Steroid therapy, Antibiotic use	III	Blood	FLC	24	ND	Died
[ 40 ]	2021	USA	F	38	SLE, HT, DS, Obesity	MV, Use of vasopressor agents, Antecedent Steroid therapy, Antibiotic use	III	Wound	FLC	32	ND	Survived
[ 40 ]	2021	USA	M	71	DS, HT, DLP	MV, use of vasopressor agents, Antecedent Steroid therapy, Antibiotic use	III	Blood	FLC	30	ND	Died
[ 40 ]	2021	USA	F	75	DS, HT, DLP	MV, use of vasopressor agents, Antecedent Steroid therapy, Antibiotic use	III	Blood	FLC	12	MFG	Survived
[ 40 ]	2021	USA	F	68	DS, bladder cancer	MV, use of vasopressor agents, Antecedent Steroid therapy, Antibiotic use	III	Urine	FLC	32	ND	Survived
[ 40 ]	2021	USA	M	65	HT	MV, use of vasopressor agents, Antecedent Steroid therapy, Antibiotic use	III	BAL	FLC	12	ND	Died
[ 40 ]	2021	USA	M	69	HT	MV, use of vasopressor agents, Antecedent Steroid therapy, Antibiotic use	III	Blood	FLC	28	MFG	Died
[ 40 ]	2021	USA	M	41	HT, CKD	MV, use of vasopressor agents, Antibiotic use	III	Blood	FLC	20	MFG	Survived
[ 40 ]	2021	USA	M	68	ND	MV, use of vasopressor agents, Antibiotic use	III	Wound	FLC	33	MFG	Survived
[ 38 ]	2021	Brazil	M	59	DVT	CVC, HD, MV, UC, Antifungal therapy, Antibiotic use	I	CVC‐tip	MDS	42	Yes	Survived
[ 38 ]	2021	Brazil	M	79	Biliary lithiasis	CVC, MV, UC, Antifungal therapy, Antibiotic use	I	CVC‐tip, Axillae, Groin, Nostrils and Ear swab	MDS	46	Yes	Survived
[ 38 ]	2021	Brazil	M	72	Stroke, dementia	CVC, MV, UC, Antifungal therapy, Antibiotic use	I	Urine	MDS	36	Yes	Died
[ 38 ]	2021	Brazil	M	58	HT, DS, obesity	CVC, Antibiotic use	I	Axillae, Groins swabs	MDS	27	No	Survived
[ 38 ]	2021	Brazil	M	63	HT, DS, Obesity	CVC, Antibiotic use	I	Axillae, Groin and nostril Swabs	MDS	18	No	Survived
[ 38 ]	2021	Brazil	F	75	HT, DS, Hypothyroidism	CVC, HD, MV, UC, Antifungal therapy, Antibiotic use	I	Axillae, Groins swabs	MDS	32	Yes	Survived
[ 38 ]	2021	Brazil	M	63	HT, DS, CKD	CVC, HD, MV, UC, Antifungal therapy, Antibiotic use	I	Axillae, groin, nostrils and ear swabs	MDS	22	Yes	Survived
[ 38 ]	2021	Brazil	M	77	COPD, Stroke, CKD	UC, Antibiotic use	I	Axillae, groin, Nostrils and Ear swabs	MDS	22	No	Survived
[ 38 ]	2021	Brazil	F	74	DS, HT, CKD, Coronary artery disease	CVC, MV, HD	I	Blood	MDS	34	Yes	Died
[ 39 ]	2021	Qatar	M	64	None	MV, Antibiotic use, HD	ND	Blood	AMB, FLC	47	ANF	Died
[ 32 ]	2021	Lebanon	M	75	ARDS ,Metastatic prostate cancer	Intubated, MV, CVC, UC, Antibiotic use, Steroid therapy, Antifungal therapy	ND	DTA, Urine, Blood	ND	40	Yes	Survived
[ 32 ]	2021	Lebanon	F	82	COPD, Respiratory failure	MV, CVC, UC, Antibiotic use, Steroid therapy, Antifungal therapy	ND	DTA	FLC, AMB	26	No	Survived
[ 32 ]	2021	Lebanon	M	68	ARDS	Intubated MV, CVC, UC, Antibiotic use, Steroid therapy, Antifungal therapy	ND	DTA	ND	50	No	Survived
[ 32 ]	2021	Lebanon	F	68	ARDS	Intubated, MV, CVC,UC, Antibiotic use, Steroid therapy, Antifungal therapy	ND	DTA	FLC, AMB	40	Yes	Survived
[ 32 ]	2021	Lebanon	M	71	Cutaneous T cell lymphoma	Intubated, MV, CVC,UC, Antibiotic use, Steroid therapy, Antifungal therapy	ND	DTA	FLC, AMB	15	Yes	Survived
[ 32 ]	2021	Lebanon	M	85	ARDS	Intubated, MV, CVC,UC Antibiotic use, Steroid therapy, Antifungal therapy	ND	DTA	ND	10	Yes	Survived
[ 32 ]	2021	Lebanon	M	79	ARDS, CLL	Intubated, MV, CVC,UC, Antibiotic use, Steroid therapy, Antifungal therapy	ND	DTA	ND	48	No	Survived
[ 37 ]	2021	Turkey	M	71	Stroke, DS, Donation of a single kidney, lobectomy surgery due to lung cancer	Favipiravir and intravenous Dexamethasone therapy, Antibiotic use	ND	Blood	AMB, FLC	ND	CAS	Died
[ 44 ]	2022	India	M	36	Hepatomegaly, Aplastic anemia, Malignancy, sHLH, HRF, ARDS, MOF, AKD	Steroid therapy, MV	ND	Blood	FLC	29	FLC, CAS	Died
[ 45 ]	2022	Germany	F	65	ARDS	Steroid therapy, MV	I	Urine, BAL	FLC, CAS	90	VRC	Survived
[ 45 ]	2022	Germany	M	60	Lung transplant, EAA, AKD	MV	I	Blood, TBS	FLC, CAS	73	AMB, CAS, MFG, POS	Survived
[ 43 ]	2022	Italy	M	64	ARDS	MV, Steroid therapy, Antibiotic use	ND	Skin	FLC	100	ANF	Survived
[ 43 ]	2022	Italy	M	64	Respiratory disease, Smoker, HTA, DS, ARDS	MV, Steroid therapy, Antibiotic use, Antifungal therapy	ND	Skin	FLC	16	No	Died
[ 43 ]	2022	Italy	F	49	Respiratory disease, HTA, DS, Autoimmune disease, ARDS	MV , Steroid therapy, Antibiotic use	ND	Skin	FLC	25	No	Died
[ 43 ]	2022	Italy	M	57	Autoimmune disease, ARDS	MV, Steroid therapy, Immunomodulatory Agents, Antibiotic use, Antifungal therapy	ND	Urine	FLC	28	No	Died
[ 43 ]	2022	Italy	F	55	HTA, Hematological disease, Malignancy, ARDS	MV, Steroid therapy, Immunomodulatory Agents, Antibiotic use, Antifungal therapy	ND	Respiratory tract, Blood	FLC	100	ANF, AMB	Survived
[ 43 ]	2022	Italy	F	58	Respiratory disease, HTA, DS, Autoimmune disease, ARDS	MV, Steroid therapy, Antibiotic use, Antifungal therapy	ND	Skin	FLC	66	No	Survived

Abbreviations: MV: mechanical ventilation, PICCs: peripherally inserted central lines, UC: urinary catheters, CLD: chronic liver disease, AKD: acute kidney disease, HT: hypertension, DS: diabetes, IHD: Ischemic heart disease, DLP: dyslipidemia, MM: multiple myeloma, SCT: stem cell transplantation, CVC: central venous catheter, COPD: chronic obstructive pulmonary disease, DVT: Deep-seated venous thrombosis, ARDS: Acute respiratory distress syndrome, HD: HD, SLE: Systemic lupus erythematosus, CAD: Coronary artery Disease, CKD: Chronic kidney disease, BSI: blood stream infection, CLL: Chronic lymphocytic leukemia, ANF: Anidulafungin, CAS: Caspofungin, MFG: Micafungin, ISA: Isavuconazole, VRC: Voriconazole, POS: posaconazole, MAR: Multiazole-resistant, Echino: Echinocandins, MDS: Multidrug-susceptible, AMB: Amphotericin B, FLC: Fluconazole, MDS: Multidrug-susceptible, sHLH: Secondary Hemophagocytic Lymphohistiocytosis, HRF: hypoxemic respiratory failure, MOF: multi-organ failure, EAA: Exogenous allergic alveolitis, TBS: Tracheo-bronchial secretion, HTA: Arterial Hypertension, DTA: Deep Tracheal Aspirates, ND: Not define

COVID-19-associated *C. auris* infection was more common in males (51/70, 72.86%) than females (19/70, 27.14%; data were not available for 5 patients). The mean±SD age of patients was 63.8±12.09 years [ 12 , 32 - 34 , 36 - 45 ]. Antibiotic use (63/68, 92.65%; data were not available for 7 patients) was the most common risk factor, followed by steroid therapy (48/68, 70.59%; data were not available for 7 patients), mechanical ventilation (48/68, 70.59%; data were not available for 7 patients), the use of urinary catheters (35/68, 51.47%; data were not available for 7 patients), central venous catheter (25/68, 36.76%; data were not available for 7 patients), peripherally inserted central lines (12/68, 17.65%; data were not available for 7 patients), and vasopressor drugs (12/68, 17.65%; data were not available for 7 patients). Regarding the comorbidities, hypertension (33/69, 47.82%; data were not available for 6 patients), diabetes mellitus (25/69, 36.23%; data were not available for 6 patients), acute respiratory distress syndrome (13/69, 18.84%; data were not available for 6 patients), and obesity (13/69, 18.84%; data were not available for 6 patients) were recorded for the patients in descending order of prevalence. 

**Figure 1:**
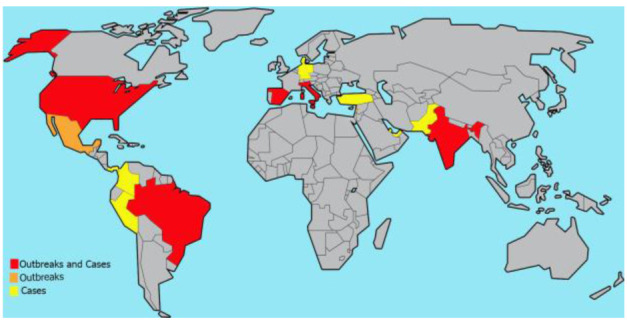
Global distribution of COVID-19-associated *C. auris* infections (cases/outbreaks) (data for Panama is extracted from the WHO epidemiological alert [ 35 ]). ADDIN

COVID-19-associated *C. auris* infections (cases/outbreaks) are not limited to a specific geographical region. As shown in Figure 1, they have been reported from American, European, and Asian countries. Lack of reports from other parts of the world does not necessarily mean a lack of such infections, but a lack of sufficient data, which indicates the need for further studies.

According to genetic traits, *C. auris* is classified in five clades [ 46 ] with different geographic distribution patterns. According to the results of the included studies, from 42 isolates with available data, 18 (42.86%), 12 (28.57%), and 12 (28.57%) were classified as clade I, III, and IV, respectively [ 12 , 36 , 38 , 40 , 41 , 45 ]. Clade I was found in Italy, Brazil, Germany, and Lebanon, while Clade III and Clade IV were mainly reported from the United States and Mexico, respectively [ 12 , 36 , 38 , 40 , 41 , 45 , 47 ].

Inter-clade difference in susceptibility pattern of *C. auris* is reported in some studies [ 48 ]. Results of the present review confirm the inter-clade difference. While all isolates of clades III and IV were resistant to at least one antifungal drug, 11 out of 18 isolates of clade I were susceptible to antifungal agents. As the available data might be still scarce to make a firm conclusion, special attention to genetic characterization of *C. auris* isolates in different studies would be beneficial in this regard and is recommended. 

Due to some features, *C. auris* is more likely to cause a hospital outbreak than other *Candida* species [ 27 , 49 , 50 ]. Biofilm formation is one of these pathogenesis traits that lead to withstanding desiccation and persistence in environments and health care settings [ 51 ]. Elongated survival on environmental surfaces and healthcare-mediated exogenous transmission between patients are other facilitating factor for this fungus. As a result, outbreaks, which continue for several months and sometimes lead to the closing of intensive care units, continuously have been described [ 33 , 52 ]. During the current pandemic, the overload of ICUs has been a breeding ground for the emergence and expansion of *C. auris* [ 12 , 17 , 34 , 38 ]. Based on our literature review, 9 COVID-19-associated *C. auris* outbreaks have been reported [ 12 , 17 , 32 - 34 , 38 , 40 , 47 , 53 ]. It is noteworthy that in some of these countries including Lebanon, Brazil, Mexico, and Peru, no isolates of this pathogen had been noted prior to this period [ 12 , 32 , 35 , 38 ]. Details of the outbreaks are presented in Table 2.

**Table 2:** Features of COVID-19-associated *C. auris* outbreaks reported up to September 12, 2022

Publication Year	Continent	Country	City/State	Patients (NO.)	Clade (I,II,III,IV,V)	Resistance Pattern	Outcome	Ref.
2020	North America	Mexico	Monterrey, Nuevo Leon	12	IV (South American)	AMB:6 , AMB+FLU: 5, AMB+ANF : 1	Survived: 4 , Died: 8	[ 12 ]
2020	Asia	India	New Delhi	10	ND	FLC: 3, FLC+AMB: 1,	FLC+5-FC: 1,FLC+VOR+AMB+5-FC: 1, FLC+ AMB+5-FC: 2, FLC+VOR+5-FC: 2	Died:6, Survived:4	[ 33 ]
2021	North America	USA	Florida	35	ND	ND	Died:8	[ 17 ]
2021	Asia	Lebanon	Beirut	7	ND	FLU+AMB: 3	Survived (Still in ICU): 7	[ 32 ]
2021	Europe	Italy	Genoa	6	ND	AMB+azoles: 6	Died: 3, Survived: 3	[ 34 ]
2021	South America	Brazil	São Paulo	9	I (South Asian)	MDR	Died:2 , Survived:7	[ 38 ]
2021	North America	USA	Miami	12	III (South African)	Echino: 1, FLC: 12	Died:6, Survived:6	[ 40 ]
2021	Europe	Spain	Valencia	56	ND	Echino: 2, FLC: 56	ND	[ 53 ]
2022	Asia	Lebanon	Beirut	32	I	AMB, FLC, VOR	Died:19, Survived:13	[ 47 ]

Abbreviations: MAR: Multiazole-resistant، Echino: Echinocandins, FLC: Fluconazole: MDS: multidrug-susceptible, ANF: Anidulafungin, VOR: Voriconazole, AMB: Amphotericin B, 5-FC: 5-ﬂucytosine, ND: Not defined

## The impact of COVID-19 on AMR

One of the unforeseen and unavoidable consequences of the COVID-19 pandemic is the appearance of antimicrobial resistance [ 54 ]. It is anticipated that too much and inappropriate use of antibiotics, disinfectants, and biocides during this pandemic may raise devastating effects on antifungal resistance control and antibiotic stewardship programs [ 15 ]. 

In the current pandemic, hospitalized patients with COVID-19 are more predisposed to superinfections with bacterial and/or fungal pathogens which is likely to impact the mortality rates [ 55 ]. This phenomenon is especially important in the case of emerging resistant species, such as *C. auris* [ 55 ]. An association between antibiotic use and the emergence of candidemia by *Candida* species with high minimum inhibitory concentration and/or intrinsic resistance to fluconazole has been reported [ 56 , 57 ]. Along the same line, up to 94% of COVID-19 hospitalized patients receive antimicrobial agents [ 58 , 59 ], which may increase the colonization rate of *Candida* species, such as *C. auris* [ 60 ]. In our literature review, results of antifungal susceptibility testing showed that 59 out of 70 (84.29%) isolates with available data were resistant to at least one antifungal drug. Among them, 31 (44.29%) isolates were multidrug resistant, which is 14.29% higher than the CDC report (30%) [ 61 ]. As shown in Table 2, in all reported COVID-19-associated *C. auris* outbreaks, drug-resistant isolates play a key role, and it makes the management more complicated. 

## Conclusion

With the increased hospital stay and the higher need for intensive care, COVID-19 patients are at risk for *C. auris* infections. Regarding the specific features of this fungus, it can circulate within clinical settings and cause outbreaks. Moreover, due to the different conditions in COVID-19 patients which are in favor of the selection of drug-resistant organisms, these patients are at risk for coinfections by single or multi-drug resistant *C. auris*. Accordingly, attempts for timely diagnosis and targeted treatment of such infections in COVID-19 patients should be made.

## Acknowledgments

None. 

## Authors’ contribution

Conceptualization: S.K., M.A., and S.M. Literature search: J.J., S.A.H, and I.H. Draft preparation: S.K., S.A.G, and S.M. Critical review: H.T., S.A.G, M.A, and S.M. All authors read and approved the final manuscript. 

## Conflicts of interest

The authors declare no competing interests.

## Financial disclosure

This research received no specific grant from any funding agency, commercial or not-for-profit sectors.

## References

[1] Hu B, Guo H, Zhou P, Shi ZL ( 2021). Characteristics of SARS-CoV-2 and COVID-19. Nat Rev Microbiol.

[2] Murray AK ( 2020). The novel coronavirus COVID-19 outbreak: global implications for antimicrobial resistance. Front Microbiol.

[3] Hashan MR, Smoll N, King C, Ockenden-Muldoon H, Walker J, Wattiaux A, et al ( 2021). Epidemiology and clinical features of COVID-19 outbreaks in aged care facilities: A systematic review and meta-analysis. EClinicalMedicine.

[4] Weston S, Frieman MB ( 2020). COVID-19: knowns, unknowns, and questions. Msphere.

[5] Wiersinga WJ, Rhodes A, Cheng AC, Peacock SJ, Prescott HC ( 2020). Pathophysiology, transmission, diagnosis, and treatment of coronavirus disease 2019 (COVID-19): a review. JAMA.

[6] Triggle CR, Bansal D, Farag EABA, Ding H, Sultan AA ( 2020). COVID-19: learning from lessons to guide treatment and prevention interventions. MSphere.

[7] Salehi M, Ahmadikia K, Badali H, Khodavaisy S ( 2020). Opportunistic fungal infections in the epidemic area of COVID-19: a clinical and diagnostic perspective from Iran. Mycopathologia.

[8] Chen N, Zhou M, Dong X, Qu J, Gong F, Han Y, et al ( 2020). Epidemiological and clinical characteristics of 99 cases of 2019 novel coronavirus pneumonia in Wuhan, China: a descriptive study. Lancet.

[9] Lai CC, Wang CY, Hsueh PR ( 2020). Co-infections among patients with COVID-19: The need for combination therapy with non-anti-SARS-CoV-2 agents?. J Microbiol Immunol Infect.

[10] Nazari T, Sadeghi F, Izadi A, Sameni S, Mahmoudi S ( 2022). COVID-19-associated fungal infections in Iran: A systematic review. PloS One.

[11] Du H, Bing J, Hu T, Ennis CL, Nobile CJ, Huang G ( 2020). Candida auris: Epidemiology, biology, antifungal resistance, and virulence. PLoS Pathog.

[12] Villanueva-Lozano H, Treviño-Rangel RJ, González GM, Ramírez-Elizondo MT, Lara-Medrano R, Aleman-Bocanegra MC, et al ( 2021). Outbreak of Candida auris infection in a COVID-19 hospital in Mexico. Clin Microbiol Infect.

[13] Vaseghi N, Sharifisooraki J, Khodadadi H, Nami S, Safari F, Ahangarkani F, et al ( 2022). Global prevalence and subgroup analyses of coronavirus disease (covid‐19) associated candida auris infections (caca): a systematic review and meta‐analysis. Mycoses.

[14] Khor WP, Olaoye O, D’arcy N, Krockow EM, Elshenawy RA, Rutter V, et al ( 2020). The need for ongoing antimicrobial stewardship during the COVID-19 pandemic and actionable recommendations. Antibiotics.

[15] Rezasoltani S, Yadegar A, Hatami B, Aghdaei HA, Zali MR ( 2020). Antimicrobial resistance as a hidden menace lurking behind the COVID-19 outbreak: the global impacts of too much hygiene on AMR. Front Microbiol.

[16] Moin S, Farooqi J, Rattani S, Nasir N, Zaka S, Jabeen K ( 2021). C. auris and non-C. auris candidemia in hospitalized adult and pediatric COVID-19 patients; single center data from Pakistan. Med Mycol.

[17] Prestel C, Anderson E, Forsberg K, Lyman M, de Perio MA, Kuhar D, et al ( 2021). Candida auris outbreak in a COVID-19 specialty care unit—Florida, July–August 2020. MMWR.

[18] Rajni E, Singh A, Tarai B, Jain K, Shankar R, Pawar K, et al ( 2021). A high frequency of Candida auris blood stream infections in COVID-19 patients admitted to intensive care units, North-western India: A case control study. Open Forum Infect Dis.

[19] Satoh K, Makimura K, Hasumi Y, Nishiyama Y, Uchida K, Yamaguchi H ( 2009). Candida auris sp. nov., a novel ascomycetous yeast isolated from the external ear canal of an inpatient in a Japanese hospital. Microbiol Immunol.

[20] Ruiz-Gaitán A, Martínez H, Moret AM, Calabuig E, Tasias M, Alastruey-Izquierdo A, et al ( 2019). Detection and treatment of Candida auris in an outbreak situation: risk factors for developing colonization and candidemia by this new species in critically ill patients. Expert Rev Anti Infect Ther.

[21] Schelenz S, Hagen F, Rhodes JL, Abdolrasouli A, Chowdhary A, Hall A, et al ( 2016). First hospital outbreak of the globally emerging Candida auris in a European hospital. Antimicrob Resist Infect Control.

[22] Hata DJ, Humphries R, Lockhart SR, Committee CoAPM ( 2020). Candida auris: an emerging yeast pathogen posing distinct challenges for laboratory diagnostics, treatment, and infection prevention. Arch Pathol Lab Med.

[23] Jeffery-Smith A, Taori SK, Schelenz S, Jeffery K, Johnson EM, Borman A, et al ( 2018). Candida auris: a review of the literature. Clin Microbiol Rev.

[24] Cadnum JL, Shaikh AA, Piedrahita CT, Sankar T, Jencson AL, Larkin EL, et al ( 2017). Effectiveness of disinfectants against Candida auris and other Candida species. Infect Control Hosp Epidemiol.

[25] Piedrahita CT, Cadnum JL, Jencson AL, Shaikh AA, Ghannoum MA, Donskey CJ ( 2017). Environmental surfaces in healthcare facilities are a potential source for transmission of Candida auris and other Candida species. Infect Control Hosp Epidemiol.

[26] Uppuluri P ( 2020). Candida auris biofilm colonization on skin niche conditions. Msphere.

[27] Eyre DW, Sheppard AE, Madder H, Moir I, Moroney R, Quan TP, et al ( 2018). A Candida auris outbreak and its control in an intensive care setting. N Engl J Med.

[28] Taghizadeh Armaki M, Mahdavi Omran S, Kiakojuri K, Khojasteh S, Jafarzadeh J, Tavakoli M, et al ( 2021). First fluconazole-resistant Candida auris isolated from fungal otitis in Iran. Curr Med Mycol.

[29] Sabino R, Veríssimo C, Pereira ÁA, Antunes F ( 2020). Candida auris, an agent of hospital-associated outbreaks: which challenging issues do we need to have in mind?. Microorganisms.

[30] Cortegiani A, Misseri G, Giarratano A, Bassetti M, Eyre D ( 2019). The global challenge of Candida auris in the intensive care unit. Crit Care.

[31] Alfonso-Sanchez JL, Agurto-Ramirez A, Chong-Valbuena MA, De-Jesús-María I, Julián-Paches P, López-Cerrillo L, et al ( 2021). The Influence of infection and colonization on outcomes in inpatients With COVID-19: are we forgetting something?. Front Public Health.

[32] Allaw F, Kara Zahreddine N, Ibrahim A, Tannous J, Taleb H, Bizri AR, et al ( 2021). First Candida auris outbreak during a COVID-19 pandemic in a Tertiary-Care Center in Lebanon. Pathogens.

[33] Chowdhary A, Tarai B, Singh A, Sharma A ( 2020). Multidrug-resistant Candida auris infections in critically ill coronavirus disease patients, India, April–July 2020. Emerg Infect Dis.

[34] Magnasco L, Mikulska M, Giacobbe DR, Taramasso L, Vena A, Dentone C, et al ( 2021). Spread of carbapenem-resistant Gram-negatives and Candida auris during the COVID-19 pandemic in critically ill patients: one step back in antimicrobial stewardship?. Microorganisms.

[35] WHO Epidemiological alert: Candida auris outbreaks in health care services. Regional Office for the Americas of the World Health Organization. 2016. https://www.paho.org/en/documents/epidemiological-alert-candida-auris-outbreaks-health-care-services-context-covid-19.

[36] de Almeida JN, Francisco EC, Hagen F, Brandão IB, Pereira FM, Presta Dias PH, et al ( 2021). Emergence of Candida auris in Brazil in a COVID-19 intensive care unit. J Fungi (Basel).

[37] Bölükbaşı Y, Erköse Genç G, Orhun G, Kuşkucu MA, Çağatay A, Önel M, et al ( 2021). First Case of COVID-19 Positive Candida auris Fungemia in Turkey. Mikrobiyol Bul.

[38] de Almeida Jr JN, Brandão IB, Francisco EC, de Almeida SLR, de Oliveira Dias P, Pereira FM, et al ( 2021). Axillary Digital Thermometers uplifted a multidrug‐susceptible Candida auris outbreak among COVID‐19 patients in Brazil. Mycoses.

[39] Goravey W, Ali GA, Ali M, Ibrahim EB, Al Maslamani M, Hadi HA ( 2021). Ominous combination: COVID‐19 disease and Candida auris fungemia—Case report and review of the literature. Clin Case Rep.

[40] Hanson BM, Dinh AQ, Tran TT, Arenas S, Pronty D, Gershengorn HB, et al ( 2021). Candida auris invasive infections during a COVID-19 case surge. Antimicrob Agents Chemother.

[41] Pilato VD, Codda G, Ball L, Giacobbe DR, Willison E, Mikulska M, et al ( 2021). Molecular epidemiological investigation of a nosocomial cluster of c. auris: evidence of recent emergence in Italy and ease of transmission during the COVID-19 Pandemic. J Fungi (Basel).

[42] Schwartz RA, Kapila R ( 2020). Cutaneous manifestations of a 21st century worldwide fungal epidemic possibly complicating the COVID‐19 pandemic to jointly menace mankind. Dermatol Ther.

[43] Corcione S, Montrucchio G, Shbaklo N, De Benedetto I, Sales G, Cedrone M, et al ( 2022). First cases of candida auris in a referral intensive care unit in piedmont region, Italy. Microorganisms.

[44] Gautam S, Sharma G, Singla S, Garg S ( 2022). Case Report: secondary hemophagocytic lymphohistiocytosis (shlh) and Candida auris Fungemia in Post-acute COVID-19 Syndrome: a clinical challenge. Front Med (Lausanne).

[45] Hinrichs C, Wiese‐Posselt M, Graf B, Geffers C, Weikert B, Enghard P, et al ( 2022). Successful control of Candida auris transmission in a German COVID‐19 intensive care unit. Mycoses.

[46] Spruijtenburg B, Badali H, Abastabar M, Mirhendi H, Khodavaisy S, Sharifisooraki J, et al ( 2022). Confirmation of fifth Candida auris clade by whole genome sequencing. Emerg Microbes Infect.

[47] Allaw F, Haddad SF, Habib N, Moukarzel P, Naji NS, Kanafani ZA, et al ( 2022). COVID-19 and C. auris: A case-control study from a Tertiary Care Center in Lebanon. Microorganisms.

[48] Chow NA, Muñoz JF, Gade L, Berkow EL, Li X, Welsh RM, et al ( 2020). Tracing the evolutionary history and global expansion of Candida auris using population genomic analyses. MBio.

[49] Heaney H, Laing J, Paterson L, Walker AW, Gow NA, Johnson EM, et al ( 2020). The environmental stress sensitivities of pathogenic Candida species, including Candida auris, and implications for their spread in the hospital setting. Med Mycol.

[50] Wickes BL ( 2020). Analysis of a Candida auris outbreak provides new insights into an emerging pathogen. J Clin Microbiol.

[51] Horton MV, Nett JE ( 2020). Candida auris infection and biofilm formation: going beyond the surface. Curr Clin Microbiol Rep.

[52] Kean R, Brown J, Gulmez D, Ware A, Ramage G ( 2020). Candida auris: a decade of understanding of an enigmatic pathogenic yeast. J Fungi (Basel).

[53] Mulet Bayona JV, Tormo Palop N, Salvador García C, Fuster Escrivá B, Chanzá Aviñó M, Ortega García P, et al ( 2021). Impact of the SARS-CoV-2 Pandemic in Candidaemia, Invasive Aspergillosis and Antifungal Consumption in a Tertiary Hospital. J Fungi (Basel).

[54] Lai CC, Chen SY, Ko WC, Hsueh PR ( 2021). Increased antimicrobial resistance during the COVID-19 pandemic. Int J Antimicrob Agents.

[55] Roudbary M, Kumar S, Kumar A, Černáková L, Nikoomanesh F, Rodrigues CF ( 2021). Overview on the prevalence of fungal infections, immune response, and microbiome role in COVID-19 patients. J Fungi (Basel).

[56] Ben-Ami R, Olshtain-Pops K, Krieger M, Oren I, Bishara J, Dan M, et al ( 2012). Antibiotic exposure as a risk factor for fluconazole-resistant Candida bloodstream infection. Antimicrob Agents Chemother.

[57] Lin MY, Carmeli Y, Zumsteg J, Flores EL, Tolentino J, Sreeramoju P, et al ( 2005). Prior antimicrobial therapy and risk for hospital-acquired Candida glabrata and Candida krusei fungemia: a case-case-control study. Antimicrob Agents Chemother.

[58] Mason KL, Erb Downward JR, Mason KD, Falkowski NR, Eaton KA, Kao JY, et al ( 2012). Candida albicans and bacterial microbiota interactions in the cecum during recolonization following broad-spectrum antibiotic therapy. Infect Immun.

[59] Romo JA, Kumamoto CA ( 2020). On commensalism of Candida. J Fungi (Basel).

[60] Arastehfar A, Carvalho A, Nguyen MH, Hedayati MT, Netea MG, Perlin DS, et al ( 2020). Covid-19-associated candidiasis (CAC): an underestimated complication in the absence of immunological predispositions?. J Fungi (Basel).

[61] Health UDo, Services H ( 2019). CDC. Antibiotic resistance threats in the United States, 2019.

